# Subcutaneous Infusion of DNA-Aptamer Raised against Advanced Glycation End Products Prevents Loss of Skeletal Muscle Mass and Strength in Accelerated-Aging Mice

**DOI:** 10.3390/biomedicines11123112

**Published:** 2023-11-22

**Authors:** Yusaku Mori, Makoto Ohara, Michishige Terasaki, Naoya Osaka, Hironori Yashima, Tomomi Saito, Yurie Otoyama-Kataoka, Takemasa Omachi, Yuichiro Higashimoto, Takanori Matsui, Tomoyasu Fukui, Sho-ichi Yamagishi

**Affiliations:** 1 Anti-Glycation Research Section, Division of Diabetes, Metabolism, and Endocrinology, Department of Medicine, Showa University School of Medicine, Shinagawa, Tokyo 142-8555, Japan; 2 Division of Diabetes, Metabolism, and Endocrinology, Department of Medicine, Showa University School of Medicine, Shinagawa, Tokyo 142-8555, Japanttmichi@med.showa-u.ac.jp (M.T.); n.oosaka@med.showa-u.ac.jp (N.O.); yurie.o.0404@med.showa-u.ac.jp (Y.O.-K.); t.omaki@med.showa-u.ac.jp (T.O.);; 3 Department of Chemistry, Kurume University School of Medicine, Kurume 830-0011, Fukuoka, Japan; higashiy@med.kurume-u.ac.jp; 4 Department of Bioscience and Biotechnology, Fukui Prefectural University, Eiheiji 910-1195, Fukui, Japan

**Keywords:** AGEs, DNA aptamer, MuRF1, muscle atrophy, oxidative stress, sarcopenia

## Abstract

We have developed DNA aptamers that can inhibit the toxic effects of advanced glycation end products (AGE-Apts). We herein evaluated the effects of AGE-Apts on muscle mass and strength in senescence-accelerated mouse prone 8 (SAMP8) mice. Eight-month-old male SAMP8 mice received subcutaneous infusion of control DNA aptamers (CTR-Apts) or AGE-Apts. Mice in an age-matched senescence-accelerated mouse resistant strain 1 (SAMR1) group were treated with CTR-Apts as controls. The soleus muscles were collected after the 8-week intervention for weight measurement and histological, RT-PCR, and immunofluorescence analyses. Grip strength was measured before and after the 8-week intervention. AGE-Apt treatment inhibited the progressive decrease in the grip strength of SAMP8 mice. SAMP8 mice had lower soleus muscle weight and fiber size than SAMR1 mice, which was partly restored by AGE-Apt treatment. Furthermore, AGE-Apt-treated SAMP8 mice had a lower interstitial fibrosis area of the soleus muscle than CTR-Apt-treated SAMP8 mice. The soleus muscle levels of AGEs, oxidative stress, receptor for AGEs, and muscle ring-finger protein-1 were increased in the CTR-Apt-treated mice, all of which, except for AGEs, were inhibited by AGE-Apt treatment. Our present findings suggest that the subcutaneous delivery of AGE-Apts may be a novel therapeutic strategy for aging-related decrease in skeletal muscle mass and strength.

## 1. Introduction

Sarcopenia is a syndrome characterized by the progressive loss of skeletal muscle mass and function [1]. It frequently occurs as a part of the aging process in older adults, which can be further facilitated by other contributing factors, including sedentary lifestyle; inadequate nutrient intake; and comorbid chronic diseases, such as diabetes, chronic kidney disease, and cancer [1,2,3,4]. Epidemiological studies have reported that sarcopenia prevalence drastically increases with aging, particularly in individuals over 75 years old; currently, sarcopenia is estimated to affect 10–27% of older adults worldwide [5].

Sarcopenia has been shown to be a major cause of disability in older adults [6], which can result in impaired quality of life. Furthermore, sarcopenia has been reportedly associated with an increased risk for several adverse health outcomes, including falls, frailty, cognitive impairment, hospitalization, and mortality [1,2,3,4]. Therefore, it has emerged as a significant public health concern in many countries, particularly those with a high aging population rate. However, to date, no pharmacological interventions have been established for sarcopenia prevention and treatment [7]. Although lifestyle modification including appropriate physical activity and optimal nutrition is currently considered a fundamental therapeutic approach for sarcopenia prevention and treatment [1], the clinical evidence of lifestyle modification is highly limited. Therefore, the development of effective pharmacological interventions against sarcopenia has long been desired.

Advanced glycation end products (AGEs) are formed through the nonenzymatic glycation of molecules, including proteins, lipids, and nucleic acids, the process of which progresses under physiological aging [8,9,10]. As AGEs are hardly degraded following their formation and slightly excluded from the kidney, they progressively accumulate in the body throughout the lifespan. An accumulating body of evidence has shown that AGEs are involved in the pathogenesis of various aging-related diseases through the interaction of cell-surface receptors for AGEs (RAGEs) via the induction of oxidative stress generation [8,9,10]. Furthermore, clinical studies including ours have reported that circulating and skin-accumulated AGE levels are associated with an increased risk of sarcopenia and subsequent frailty [11,12,13,14]. These observations led us to speculate that AGE inhibition could be a potential therapeutic target against sarcopenia.

As AGEs have been considered a promising target for therapeutic intervention in various diseases, a large number of compounds have been proposed as AGE formation inhibitors or AGE–RAGE interaction blockers [15]. However, owing to their limited efficacy or potential adverse side effects in vivo, none of these compounds have reached clinical application [15]. DNA aptamers are short single-stranded DNA sequences that can selectively bind to target molecules [16]. Compared with protein antibodies, DNA aptamers have several advantages, including short generation time, low costs of manufacturing, no batch-to-batch variability, and high modifiability and thermal stability. RNA aptamers that can inhibit vascular endothelial growth factors are clinically approved for the treatment of age-related macular degeneration, and a number of aptamers have entered clinical trials, including for ocular diseases, hematologic diseases, and cancer [16]. Recently, we have innovatively developed DNA aptamers raised against AGEs (AGE-Apts) that can inhibit the toxic effects of AGEs [17,18,19,20,21,22]. AGE-Apts bind to AGEs with high affinity and subsequently inhibit their interaction with RAGE in vitro. Furthermore, we have demonstrated that the administration of AGE-Apts prevented the development and progression of AGE-related diseases in various animal models, such as diabetic retinopathy and nephropathy, vascular remodeling following angioplasty, high-fructose-induced metabolic derangements, and tumor growth and metastasis [17,18,19,20,21,22]. However, the effects of AGE-Apts on aging-related sarcopenia remain largely unknown. This study aimed to investigate whether and how AGE-Apts could attenuate the decrease in skeletal muscle mass and strength in a mouse model of aging-accelerated sarcopenia.

## 2. Materials and Methods

### 2.1. Preparation of AGE-Apts

Control DNA aptamers (CTR-Apts) and AGE-Apts were synthesized through polymerase chain reaction (PCR) as previously described [21]. CTR-Apt and AGE-Apt sequences included 5′-aTcgAccTggAggcgAgcAgcTcggATccAgTcgcgTgAg-3′ and 5′-tgTAgcccgAgTATcATTcTccATcgcccccAgATAcAAg-3′, respectively. Phosphorothioate nucleotides are indicated as capital letters. The predicted secondary structure of AGE-Apts is presented in Supplementary Figure S1, and the characteristics and kinetics of AGE-Apts were described in detail in our previous studies [17,18,19].

### 2.2. Animal Experiments

The design of animal experiments was approved by the Animal Care Committee of Showa University School of Medicine (Approval number: 04001, Approval date: 31 March 2022). The experiments adhered to ARRIVE 2.0 guidelines and the Guide for the Care and Use of Laboratory Animals as previously described [23,24,25].

The senescence-accelerated mouse prone 8 (SAMP8) strain is widely used as a mouse model of aging-accelerated sarcopenia [26,27], and the senescence-accelerated mouse resistant strain 1 (SAMR1) strain is used as a control of the SAMP8 strain owing to their common genetic background. Eighteen male mice of the SAMP8 strain were purchased from Sankyo Labo Service (Edogawa, Tokyo, Japan) under the approval of the Society of Senescence-Accelerated Mouse Research. SAMP8 mice were divided into either the CTR-Apt or AGE-Apt group (*n* = 9 mice per group). Two mice in the AGE-Apt group were excluded from the present experiments owing to the development of a skin rash. As controls, six age-matched male mice of the SAMR1 strain were also purchased from the same laboratory. SAMR1 mice were assigned to CTR-Apt treatment to compare with SAMP8 mice in the same conditions.

At 8 months old, CTR-Apt or AGE-Apt treatment was initiated in mice by using osmotic pumps (Alzet, Cupertino, CA, USA; Model 1004), which can continuously release aptamers at the rate of 10 pmol/g body weight/day for 30 days. The AGE-Apt dosage was determined on the basis of our previous studies [16,17,18,19]. Osmotic pumps were implanted in the dorsal skin of mice and replaced after 4 weeks of the implantation. Before and after the 8-week intervention, forelimb grip strength testing was performed using a grip strength meter (Muromachi Kikai, Chuo, Tokyo, Japan; MODEL MK-380Si) according to a previous study [28]. In brief, mice were allowed to grasp the fence mounted on the force gauge using their forepaws. Subsequently, an inspector pulled their tails, and tension was recorded at the time the forepaws were released from the fence. An individual value was obtained from the average of consecutive three- to six-time measurements. To minimize technical errors among inspectors, a single inspector (T.S.) performed all the measurements in the presence of another inspector (Y.M.). At the end of the experiments, all mice were sacrificed after 6 h fasting via isoflurane overdose to collect blood, urine, and tissue samples. Urine samples were collected through bladder puncture. Since the bladder was empty in some mice, the final number of urine samples was four per group. Bilateral soleus muscles were weighed following careful isolation, and the left one was immersed in 4% paraformaldehyde for histological and immunofluorescence analyses, whereas the right one was snap-frozen for reverse transcription PCR (RT-PCR) analysis.

### 2.3. Measurement of Plasma Samples and Blood Pressure

Plasma glucose and lipid levels were determined using enzyme electrode (NIPRO, Osaka, Osaka, Japan; NIPRO stat strip) and colorimetric (Fuji Film WAKO, Osaka, Osaka, Japan) assays, respectively. Plasma AGE levels were measured using an enzyme-linked immunosorbent assay (ELISA) as previously described [19]. Urinary creatinine and 8-hydroxy-2′-deoxyguanosine (8-OHdG) levels were assessed using a colorimetric assay (Serotec, Chiyoda, Tokyo, Japan) and ELISA (Nikken Seil, Fukuroi, Shizuoka, Japan; High sensitive 8-OHdG Check ELISA; Product ID: KOG-HS10/E), respectively. Urinary 8-OHdG levels were adjusted with corresponding urinary creatinine levels according to the manufacturer’s instructions. Systolic blood pressure and pulse rates were measured a few days before the end of the experiments using a noninvasive tail-cuff method (Muromachi Kikai, Chuo, Tokyo, Japan; Model MK-2000ST) as previously described [25].

### 2.4. Assessment of Muscle Fiber Number, Size, and Interstitial Fibrosis

Following fixation in 4% paraformaldehyde for 24 h, the left soleus muscles were embedded into paraffin blocks, and cross sections were obtained from the middle part of the soleus muscles. The cross sections were stained with hematoxylin and eosin for muscle fiber number and size assessment and Masson’s trichrome for interstitial fibrosis assessment. Stained cross sections were digitized using a confocal microscope (Keyence, Osaka, Osaka, Japan; BZ-X710 microscope) and analyzed using ImageJ software version 1.53t (National Institutes of Health, Bethesda, MD, USA).

### 2.5. Immunofluorescence Staining

Immunofluorescence staining was performed as previously described [25]. In brief, the cross sections of the soleus muscles were incubated with anti-AGEs antibody (raised in rat, 1: 100) [19], anti-8-OHdG antibody (Nikken Seil, Product ID: MOG-100P, RRID: AB_1106819, raised in mouse, 1:200), anti-RAGE antibody (Santa Cruz Biotechnology, Dallas, TX, USA; Product ID: sc-365154, RRID: AB_10707685, raised in mouse, 1:50), or anti-muscle ring-finger protein 1 (Murf1) antibody (Santa Cruz Biotechnology, Product ID: sc-398608, RRID: AB_2819249, raised in mouse, 1:50) at 4 °C overnight and subsequently incubated with secondary antibodies at room temperature for 4 h. Immunofluorescence images were obtained using a confocal microscope (Keyence, Osaka, Japan; BZ-X710 microscope) and analyzed using ImageJ software version 1.53t (National Institutes of Health, Bethesda, MD, USA).

### 2.6. Real-Time RT-PCR

Total RNA was extracted from the right soleus muscles to synthesize cDNA, and quantitative real-time RT-PCR was performed using the TaqMan gene expression assay and sequence detection system (Life Technologies Japan, Minato, Tokyo, Japan; StepOne Plus) as previously described [25]. The following pre-designed TaqMan probe sets were used: *Myosin heavy-chain 1* (*Myh1*), Mm01332489_ml; *Myh2*, Mm01332564_ml; *Myh4*, Mm01332541_m1; *Myh7*, Mm00600555_m1; *Murf1*, Mm01185221_m1; *atrogin-1*, Mm00499523_m1; *myostatin*, Mm01254559_m1; *myogenic differentiation 1* (*Myod1*), Mm00440387_m1; *paired box 7* (*Pax7*), Mm01354484_m1; *myogenin*, Mm00446194_m1; *interleukin-6* (*Il-6*), Mm00446190_m1; and *monocyte chemotactic protein-1* (*Mcp-1*), Mm00441242_m1, 18S ribosomal RNA (Mm03928990_g1). The gene expression levels of 18S ribosomal RNA were used to normalize those of target molecules [29].

### 2.7. Statistics

Data were expressed as means ± standard deviations (SDs). Statistical comparison was performed using JMP software version 13 (SAS Institute Inc., Cary, NC, USA). Assuming the randomness is normally distributed, comparisons of three or more groups were tested using one-way analysis of variance (ANOVA) or two-way repeated measurement ANOVA followed by Tukey’s test as appropriate. The number of mice was decided as follows: comparison type, unpaired two-sided *t*-test; α error, 5%; β error, 20%; estimated mean difference, 15%; and estimated SD of each group, 25%. The significance level was defined as *p* < 0.05.

## 3. Results

### 3.1. AGE-Apts Inhibited the Decreases in Grip Strength and Soleus Muscle Mass in SAMP8 Mice

The SAMP8 strain is widely used as a mouse model of geriatric diseases, including sarcopenia [26,27]. Eight-month-old male SAMP8 mice received subcutaneous infusion of CTR-Apts or AGE-Apts for 8 weeks. Age-matched male mice of SAMR1, a control of the SAMP8 stain owing to their common genetic background, were treated with CTR-Apts for the same duration as controls. Anthropometric and biochemical parameters are shown in Table 1. Data were obtained following the 8-week intervention, except for initial body weights and food intakes. CTR-Apt-treated SAMR1 mice had significantly higher initial and final body weights and plasma total cholesterol levels than CTR-Apt- and AGE-Apt-treated SAMP8 mice. CTR-Apt- and AGE-Apt-treated SAMP8 mice tended to have higher plasma AGE levels than CTR-Apt-treated SAMR1 mice (*p* = 0.14 and *p* = 0.18, respectively). Compared with CTR-Apt-treated SAMR1 mice, the urinary levels of 8-OHdG, an oxidative stress marker, had a tendency (*p* = 0.07) to increase in CTR-Apt-treated SAMP8 mice, which was inhibited by AGE-Apt treatment (*p* = 0.12). The other anthropometric and biochemical parameters including food intake, pulse rate, systolic blood pressure, and plasma glucose and triglyceride levels were comparable among the three groups.

**Figure 1:**
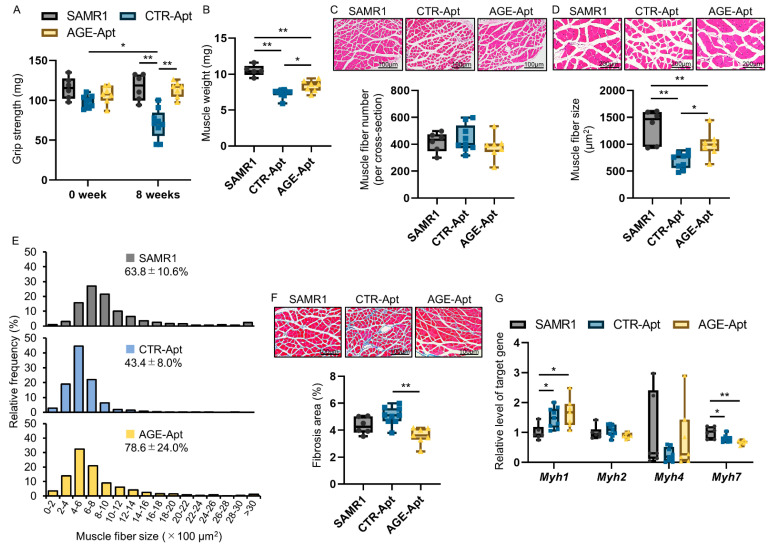
Effects of AGE-Apts on muscle function and mass in SAMP8 mice. SAMP8 mice were treated with CTR-Apts or AGE-Apts for 8 weeks. SAMR1 mice were treated with CTR-Apts as controls. (**A**) Grip strength before and after the 8-week intervention. (**B**) Soleus muscle weight following the 8-week intervention. (**C**,**D**) Muscle fiber number (**C**) and size (**D**) of the soleus muscles following the 8-week intervention. Upper panels show the representative images of the soleus muscles stained with hematoxylin and eosin. (**E**) Soleus muscle fiber size distribution. Values show coefficient of variation in soleus muscle fiber size distribution. (**F**) Interstitial fibrosis area of the soleus muscles following the 8-week intervention. Upper panels show the representative images of the soleus muscles stained with Masson’s trichrome. Magnification: (**C**) ×100; (**D**) ×200; and (**E**) ×100. (**G**) Gene expression levels of myosin heavy-chain isoforms. The data are shown as relative levels to the SAMR1 group. SAMR1, CTR-Apt-treated SAMR1; CTR-Apt, CTR-Apt-treated SAMP8; AGE-Apt, AGE-Apt-treated SAMP8. SAMR1, *n* = 6; CTR-Apt, *n* = 9; AGE-Apt; *n* = 7. * *p* < 0.05, ** *p* < 0.01.

Grip strength was measured before and after the 8-week intervention. The changes in grip strength are shown in Figure 1A. No significant difference in grip strength was noted among the three groups at baseline. However, grip strength was significantly decreased in CTR-Apt-treated SAMP8 mice following the 8-week intervention, whereas it was retained in CTR-Apt-treated SAMR1 mice. The 8-week AGE-Apt treatment significantly inhibited the decrease in the grip strength of SAMP8 mice. Following the 8-week intervention, muscle weights and muscle fiber sizes of the soleus muscles were assessed. As shown in Figure 1B–D, CTR-Apt-treated SAMP8 mice had significantly lower muscle weights and muscle fiber sizes of the soleus muscles than CTR-Apt-treated SAMR1 mice. Soleus muscle fiber size distribution is shown in Figure 1E. CTR-Apt-treated SAMP8 mice had a significantly lower coefficient of variation in the soleus muscle fiber size distribution than CTR-Apt-treated SAMR1 (*p* < 0.05). These changes in the soleus muscles of SAMP8 mice were partly restored by AGE-Apt treatment (Figure 1B–E: *p* < 0.01). Moreover, AGE-Apt-treated SAMP8 mice had a significantly lower interstitial fibrosis area of the soleus muscle than CTR-Apt-treated SAMP8 mice (Figure 1F). As shown in Figure 1G, the gene expression levels of *Myh1*, an isoform contained in fast-twitch muscle fibers [30], and *Myh7*, an isoform contained in slow-twitch muscle fibers [30], were significantly increased and decreased in the soleus muscles of CTR-Apt-treated SAMP8 mice, respectively, whereas those of *Myh2* and *Myh4*, other isoforms contained in fast-twitch muscle fibers [30], were comparable. AGE-Apt treatment did not affect the gene expression levels of these molecules in the soleus muscles (Figure 1G).

### 3.2. AGE-Apts Suppressed Oxidative Stress Generation and RAGE and MuRF1 Upregulation in the Soleus Muscles of SAMP8 Mice

**Figure 2:**
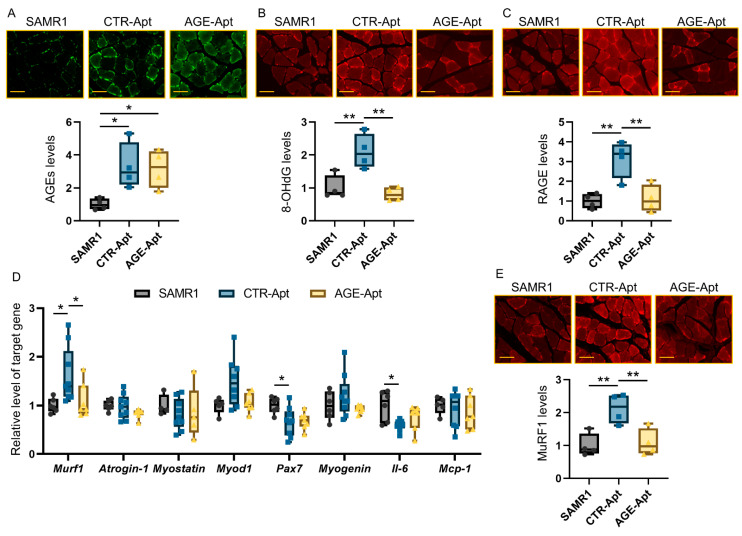
Effects of AGE-Apts on the AGE–RAGE axis in the soleus muscles of SAMP8 mice. Expression levels of AGEs (**A**), 8-OHdG (**B**), and RAGE (**C**) in the soleus muscles. (**D**) Gene expression levels of molecules related to muscle degradation, regeneration, and inflammation in the soleus muscles. (**E**) MuRF1 protein levels in the soleus muscles. (**A**–**C**,**E**): Upper panels show the representative images of immunofluorescence staining. Magnification, ×400; bars, 50 μm. SAMR1, CTR-Apt-treated SAMR1; CTR-Apt, CTR-Apt-treated SAMP8; AGE-Apt, AGE-Apt-treated SAMP8. (**A**–**C**,**E**): *n* = 4 per group. D: SAMR1, *n* = 4–6; CTR-Apt, *n* = 8–9; AGE-Apt, *n* = 5–7. The data are shown as relative levels to the SAMR1 group. * *p* < 0.05, ** *p* < 0.01.

Next, we further evaluated the effects of AGE-Apts on the AGE–RAGE–oxidative stress axis in the soleus muscles. CTR-Apt-treated SAMP8 mice had significantly higher AGE levels in the soleus muscles than CTR-Apt-treated SAMR1 mice (Figure 2A), which was associated with the increased levels of 8-OHdG and RAGE (Figure 2B,C). Among molecules associated with muscle degradation, regeneration, and inflammation, the gene expression levels of *Murf1* were increased and those of *Pax7* and *Il-6* were decreased in the soleus muscles of CTR-Apt-treated SAMP8 mice compared with those in the CTR-Apt-treated SAMR1 mice (Figure 2D). No difference was observed in the expression levels of *atrogin-1*, *myostatin*, *MyoD1*, *myogenin*, or *Mcp-1*. Although AGE-Apt treatment did not affect the levels of AGE accumulation in the soleus muscles of SAMP8 mice (Figure 2A), it significantly decreased the levels of 8-OHdG and RAGE (Figure 2B,C). Furthermore, AGE-Apt treatment significantly suppressed the gene and protein expression levels of MuRF1 in the soleus muscles of SAMP8 mice (Figure 2D,E).

## 4. Discussion

Sarcopenia is a major cause of disability in older adults, which is also associated with various adverse health outcomes, including mortality [1,2,3,4]. As sarcopenia prevalence is reportedly high in the older adult population [5], it has emerged as a major public health concern in many countries. However, effective pharmacological interventions for the prevention and treatment of aging-associated sarcopenia have not yet been established.

Although several pathways and mechanisms underlying aging-related sarcopenia have been proposed, the main one that can be a target of pharmacological interventions is yet to be elucidated. Clinical studies have reported that AGEs are associated with an increased risk of sarcopenia [11,12,13,14]. We have previously observed that serum AGE levels are significantly increased according to the frailty status of patients undergoing dialysis and inversely associated with physical performance and activity in these patients. Furthermore, we have shown that AGE-Apt treatment could prevent muscle fiber atrophy and mitochondrial dysfunction in the gastrocnemius muscle in a mouse model of chronic kidney disease [4]. However, the effects of AGE-Apts on aging-related sarcopenia remain largely unknown. SAMP8 mice represent accelerated aging with a shortened life span, thereby being widely used as a mouse model of geriatric diseases, including sarcopenia [26,27]. Therefore, in the present experiments, we have evaluated the effects of AGE-Apts on aging-accelerated sarcopenia in SAMP8 mice. Consistent with a previous study [26], we noted that SAMP8 mice exhibited progressive weakness of grip strength, a clinical marker of skeletal muscle function for the diagnosis of sarcopenia [6]. Furthermore, SAMP8 mice showed soleus muscle atrophy as measured using the muscle mass and fiber size, which were accompanied with muscular AGE accumulation. Furthermore, we demonstrated here that the 8-week subcutaneous infusion of AGE-Apts inhibited the grip strength weakness and soleus muscle atrophy in SAMP8 mice. Considering that chronic kidney disease is characterized by accelerated aging [31], our present observations could demonstrate the active involvement of AGEs in aging-accelerated sarcopenia, suggesting that subcutaneous AGE-Apt delivery is a potential therapeutic option for aging-induced sarcopenia prevention.

AGEs elicit oxidative stress generation through interaction with RAGE in various types of cells [8,9,10], which subsequently activates redox-sensitive transcription factors, thereby leading to involvement in the pathogenesis of many diseases, including diabetic complications, cardiovascular disease, some types of cancer, and Alzheimer’s disease. Furthermore, we have previously shown that AGEs can upregulate RAGE expression via oxidative stress, thereby resulting in a positive feedback loop in the AGE–RAGE axis [32]. In the present study, we noted that AGE-Apt treatment decreased oxidative stress and RAGE expression levels in the soleus muscles of SAMP8 mice, whereas it did not affect muscular AGE accumulation. These findings indicate that AGE-Apts can inhibit the AGE–RAGE axis by working as a blocker of the binding of AGEs to RAGE, but not as an inhibitor of AGE formation including AGE–collagen cross-linking. Several studies have reported that increased oxidative stress levels could be associated with aging-induced muscle atrophy in humans and rodents [33,34,35,36]. Furthermore, muscle-specific inhibition of an antioxidative enzyme, aldehyde dehydrogenase 2, has been shown to accelerate skeletal muscle atrophy in mice, although it can be prevented through antioxidant supplementation [37]. These findings suggest that by suppressing the AGE–RAGE–oxidative stress axis in SAMP8 mice, AGE-Apts could inhibit the decrease in soleus muscle strength and mass.

Skeletal muscle mass is tightly controlled by a balance between protein synthesis and protein degradation systems [38,39]. Among protein degradation systems, ubiquitin-mediated proteasomal degradation is the major proteolytic pathway in skeletal muscles. MuRF1 and atrogin-1 are the two muscle-specific E3 ubiquitin ligases, which can activate the ubiquitin–proteasome pathway [38,39]. MuRF1 and atrogin-1 are upregulated in atrophied skeletal muscles in numerous kinds of animal models with diabetes, cancer, renal failure, immobilization, and denervation [40,41,42], indicating that MuRF1 and atrogin-1 could be molecular targets for pharmacological intervention to prevent skeletal muscle atrophy. However, the role of MuRF1 and atrogin-1 in skeletal muscle atrophy could differ depending on the background conditions. Genetic deletion of MuRF1 prevents skeletal muscle loss in aging or glucocorticoid-treated mice [43,44], whereas that of atrogin-1 cannot attenuate skeletal muscle loss in such models [43,45]. Moreover, MuRF1 and atrogin-1 expressions could be regulated by different pathways. A previous study has reported that oxidative stress elicited by H_2_O_2_ treatment can upregulate MuRF1 but not atrogin-1 expression in cultured myotubes of patients with chronic obstructive pulmonary disease [46]. In the present study, we noted that MuRF1 expression was upregulated in the atrophied soleus muscles of SAMP8 mice, which was inhibited by AGE-Apt treatment, whereas atrogin-1 expression remained unchanged irrespective of AGE-Apt treatment. Therefore, our present study suggests that MuRF1 could be upregulated by the AGE–RAGE–oxidative stress axis in the soleus muscles; MuRF1 suppression may be one of the molecular mechanisms through which AGE-Apt prevents decreases in soleus muscle mass in our model.

In this study, we observed that AGE-Apt treatment sufficiently suppressed the increased oxidative stress levels and RAGE and MuRF1 upregulation in the soleus muscles of SAMP8 mice. However, AGE-Apt treatment did not completely restore the soleus muscle weight and fiber size, suggesting the involvement of other pathways. In addition to MuRF1 and atrogin-1, we also evaluated the gene expression levels of molecules related to muscle degradation, regeneration, and inflammation, including myostatin, MyoD1, Pax7, myogenin, IL-6, and MCP-1 in the soleus muscles [47,48]. Among these molecules, the gene expression levels of *Pax7* and *Il-6* were significantly decreased in the soleus muscles of SAMP8 mice, and these changes were not attenuated by AGE-Apt treatment. Pax7 is a transcriptional factor in satellite cells, which are the precursor cells of skeletal muscles [48,49]. Pax7 has been shown to play a significant role in skeletal muscle regeneration by regulating satellite cell proliferation and differentiation, thereby acting protectively against skeletal muscle atrophy. Therefore, Pax7 downregulation via a pathway that is independent of the AGE–RAGE–oxidative stress axis may be involved in soleus muscle atrophy in our model.

In contrast to Pax7, the role of IL-6 in skeletal muscle atrophy development is controversial [49]. IL-6 is a cytokine that can exert pleiotropic effects on various types of tissues and organs, including the skeletal muscles [50]. Furthermore, skeletal muscle cells can produce and release IL-6 in response to various stimuli, including exercise, which can subsequently act on skeletal muscle cells via autocrine mechanisms [50]. IL-6 signaling induces satellite cell proliferation via signal transducers and activators of transcription 3 activation, which can contribute to skeletal muscle regeneration and hypertrophy [51]. Conversely, exposure to excessive IL-6 levels, which can be caused by inflammatory disease and cancer, has been reported to promote skeletal muscle atrophy through reduced ribosomal protein S6 kinase beta-1 phosphorylation, increased transcription of the suppressor of cytokine signaling 3 and ubiquitin ligase E3 α-II, and/or Janus kinase/STAT pathway activation [51]. Therefore, it remains unclear whether IL-6 downregulation may play a causal role in soleus muscle atrophy development, or it may occur merely as a consequence of a decreased number of soleus muscle cells or impaired exercise capacity in our model.

Alternatively, soleus muscle atrophy may have already existed in the 8-month-old SAMP8 mice when the AGE-Apt treatment started. As a previous study has reported that the grip strength of SAMP8 mice begins to decline after they turn 8 months old [27], we designed the present study to initiate the subcutaneous infusion of AGE-Apt in 8-month-old SAMP8 mice. However, a further study is needed to elucidate whether AGE-Apt treatment initiated at earlier timepoints can completely prevent soleus muscle atrophy in SAMP8 mice.

This study had several limitations. First, this study lacks data from untreated mice of both strains (SAMP8 and SAMR1) as controls to evaluate the toxic effects of aptamers. Second, skeletal muscle fiber types are classified into slow-twitch and fast-twitch [52]. A previous study has reported that skeletal muscle atrophy of SAMP8 mice is more prominent in the soleus muscles that are mainly composed of slow-twitch muscle fibers than in the extensor digitorum longus muscles that are mainly composed of fast-twitch muscle fibers [27]. Therefore, to evaluate the effects of AGE-Apt on skeletal muscle atrophy, we chose the soleus muscles in this study. However, in the skeletal muscles that are composed of both slow- and fast-twitch muscle fibers, fast-twitch ones are reported to be more prone to atrophy than slow-twitch ones in SAMP8 mice and other animal models of sarcopenia [26,53]. Additionally, such different responses of the muscle fiber types can also be observed in aging-associated sarcopenia of humans [54]. In this study, we found that in the soleus muscles of SAMP8 mice, gene expression levels of *Myh1*, an isoform contained in fast-twitch muscle fibers [30], were increased, whereas those of *Myh7*, an isoform contained in slow-twitch muscle fibers [30], were decreased. However, we only performed the experiments using the soleus muscles, which were mainly composed of slow-switch muscle fibers. Therefore, it is entirely unknown whether the roles of AGEs in skeletal muscle atrophy can vary between different muscle fiber types. Understanding the different responses of slow and fast myofibers due to intrinsic and extrinsic factors may provide insights into the molecular mechanisms underlying sarcopenia and AGE-Apt treatment. Third, AGE-Apts were chronically infused into mice using osmotic pumps implanted in the subcutaneous tissue. However, drug delivery through chronic subcutaneous infusion can be a big obstacle for the clinical application of AGE-Apts. In the previous study, we evaluated the kinetics of AGE-Apts in wild-type mice [19]. Following the administration of [γ-^32^P]ATP-labeled AGE-Apts for 7 days via the intraperitoneal route, AGE-Apts were mainly distributed in the kidney, aorta, and muscle, wherein AGE-Apt concentrations were approximately fivefold higher than those in the blood. Additionally, the elimination half-lives of AGE-Apts in the kidney were approximately 7 days. Therefore, an intermittent subcutaneous injection of AGE-Apts, such as once weekly, may inhibit the decrease in grip strength and soleus muscle mass. However, a further study must validate this assumption. Fourth, to determine molecular mechanisms underlying the effects of AGE-Apts on skeletal muscle atrophy, we evaluated the muscular expression of molecules related to muscle degradation, regeneration, and inflammation. However, previous studies have reported that other mechanisms such as mitochondrial dysfunction and impaired protein synthesis can contribute to skeletal muscle atrophy [1,6,47]. Therefore, whether these mechanisms may play a role in the soleus muscle atrophy development of our model remains unclear. Finally, although prevention of skeletal muscle atrophy and dysfunction is one of the important clinical targets, we did not evaluate the effects of AGE-Apts on SAMP8 mice survival. Therefore, it would be helpful to investigate whether AGE-Apt treatment can improve survival in rodent models of sarcopenia.

## 5. Conclusions

The subcutaneous infusion of AGE-Apts could inhibit the decrease in grip strength and soleus muscle mass by suppressing the AGE–RAGE–oxidative stress axis in a mouse model of aging-accelerated sarcopenia. A muscle-specific E3 ubiquitin ligase, MuRF1, could be upregulated by the AGE–RAGE–oxidative stress axis in the soleus muscles, and MuRF1 suppression may be one of the molecular mechanisms through which AGE-Apts prevent decreases in soleus muscle mass. Our present findings suggest that the subcutaneous delivery of AGE-Apts is a novel therapeutic strategy for aging-related decrease in skeletal muscle mass and strength.

## Figures and Tables

**Table 1:** Anthropometric and biochemical parameters of CTR-Apt-treated SAMR1, CTR-Apt-treated SAMP8, and AGE-Apt-treated SAMP8 mice.

	SAMR1	CTR-Apt	AGE-Apt
Number	6	9	7
Food intake (g/day)	7.0 ± 0.9	6.2 ± 1.0	6.3 ± 0.4
Initial body weight (g)	36.6 ± 1.7	30.1 ± 2.5 **	31.0 ± 3.2 **
Final body weight (g)	37.9 ± 2.2	29.8 ± 2.0 **	30.0 ± 1.7 **
Pulse rate (/min)	707 ± 44	662 ± 75	675 ± 76
Systolic blood pressure (mmHg)	121 ± 14	123 ± 13	117 ± 13
Plasma glucose (mg/dL)	150 ± 20	129 ± 31	125 ± 21
Plasma total cholesterol (mg/dL)	65 ± 12	29 ± 9 **	40 ± 12 **
Plasma triglycerides (mg/dL)	69 ± 14	60 ± 29	57 ± 9
Plasma AGEs (μg/mL)	0.5 ± 0.4	1.5± 1.2	1.6 ± 0.9
Urinary 8-OHdG (ng/g creatinine)	3.9 ± 0.1	13.0 ± 1.0	0.4 ± 0.1

Mean ± standard deviation (SD). SAMR1, CTR-Apt-treated SAMR1; CTR-Apt, CTR-Apt-treated SAMP8; AGE-Apt, AGE-Apt-treated SAMP8. Urinary 8-OHdG; *n* = 4 mice per group. ** *p* < 0.01 versus the CTR-Apt-treated SAMR1 group.

## Data Availability

The data presented in this study are available on request from the corresponding author.

## Supplementary Materials

The following supporting information can be downloaded at: https://www.mdpi.com/article/10.3390/biomedicines11123112/s1, Figure S1: Predicted secondary structure of AGE-Apt.
