# Supplementary figures and images for: Subcutaneous Infusion of DNA-Aptamer Raised against Advanced Glycation End Products Prevents Loss of Skeletal Muscle Mass and Strength in Accelerated-Aging Mice
Source: Biomedicines. 2023 Nov 22;11(12):3112. doi: 10.3390/biomedicines11123112 (PMC10740860; doi:10.3390/biomedicines11123112)

**Supplemental Figure S1. Predicted secondary structure of AGE-Apt.**

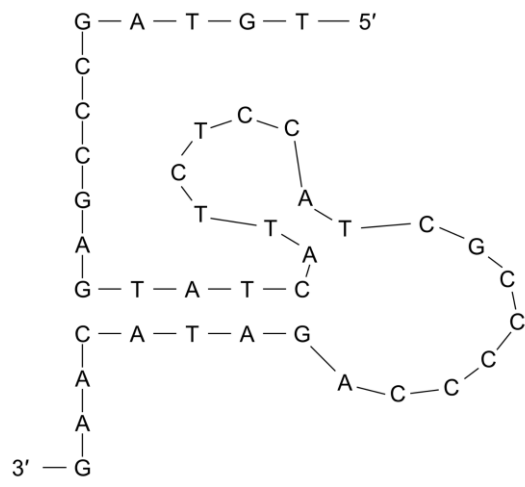

Supplement: Supplementary file 1 [file biomedicines-11-03112-s001.zip › biomedicines-2716767-supplementary.pdf]
